# Extracts from a European Letter to the Medical Record

**Published:** 1867-09

**Authors:** F. J. Bumstead


### Extracts from a European Letter to the Medical Record.

FROM PROF. F. J. BUMSTEAD.

Personal interviews with the two physicians just named [Rollet and Diday,] were most agreeable. Diday I should judge to be about 54 years of age. He looks not unlike our friend, Dr. A. C. Post, and has all the activity and vivacity of the latter. He was much interested to know whether anything was doing in America with regard to the prophylaxis of venereal, a subject which at present is engaging renewed attention, both in France and England. A novel and rather questionable idea advanced by Diday is the parasitic origin of all venereal diseases, including gonorrhœa. His argument is this: It is the prerogative of organized beings to produce their like; venereal and other contagious diseases reproduce themselves, and hence must be of parasitic origin. Diday expects that this view will yet be confirmed by the microscope. * *

Arriving in Paris a few days afterwards, of course I made it one of my first objects to see Ricord, the Nestor of the syphilitic world, and one of the few bright lights of twenty years ago still remaining. I was told that I should be likely to find him the least occupied at about eleven o'clock in the evening, when his office hours, which commenced at four in the afternoon, were *nearly* over. Calling at this hour, however, I found his waiting-room still filled with patients, and he afterwards told me that he was rarely through before twelve or one o'clock. Ricord has "aged," as an Englishman would say, since sixteen years ago, but his activity and endurance may be inferred from the amount of work of which he is still capable. If any medical man ever had reason to be satisfied with the well-merited honors that all confer upon him in his green old age, it is certainly he. There was no time to talk over with him any of the mooted questions of syphilis, but there is no doubt (and my friend, Dr. Atlee, of Philadelphia, will please notice the fact,) that Ricord now admits in full the recent doctrines upon veneral diseases, including the duality of virus, the contagiousness of the secretions of secondary lesions and the blood, and also vaccinal syphilis. Such was the universal testimony of his friends in Paris, and I afterwards heard the same from Mr. Acton, in London, who had recently spent several days with Ricord, and had freely conversed with him upon these topics. * * * * *

From Fournier's lecture-room I went into Maissonneuve's wards, where I found him making his visit and surrounded by a crowd of students. On being introduced to him, he stopped to compliment the success of American Surgery, and, among several instances, mentioned the remarkable results obtained in ovariotomy as compared with the results of the operation in Paris. I suggested that the atmosphere of hospitals was peculiarly unfavorable to this operation, but I soon found that I had touched him in a tender spot, for he immediately began a lecture lasting at least twenty minutes, in which he attempted to show that atmospheric and local influences have nothing whatever to do with the success or failure of any operation, and that everything depends upon the skill of the surgeon! "When we know how to operate in cases of ovariotomy as well as American surgeons," said he, "we shall have equally as good results." One of the internes whispered to me that this was a favorite idea of Maissonneuve, and that I had better not reply to his arguments if I wanted to see anything of his service, since he would keep on talking all day. Maissonneuve is as fond of using the *ecraseur* and his *urethrotome* as ever.—*Boston Medical Journal.*

